# Out-of-plane polarization reversal and changes in in-plane ferroelectric and ferromagnetic domains of multiferroic BiFe_0.9_Co_0.1_O_3_ thin films by water printing

**DOI:** 10.1038/s41598-023-34386-3

**Published:** 2023-05-04

**Authors:** Takuma Itoh, Kei Shigematsu, Takumi Nishikubo, Masaki Azuma

**Affiliations:** 1 Laboratory for Materials and Structures, Institute of Innovative Research, Tokyo Institute of Technology, Yokohama, 226-8503 Japan; 2 Present Address: Research Center for Magnetic and Spintronics Materials, National Institute for Materials Science, Tsukuba, 305-0047 Japan; 3 Kanagawa Institute of Industrial Science and Technology, Ebina, 243-0435 Japan; 4 Living Systems Materialogy Research Group, International Research Frontiers Initiative, Tokyo Institute of Technology, Yokohama, 226-8501 Japan

**Keywords:** Ferroelectrics and multiferroics, Structure of solids and liquids, Surfaces, interfaces and thin films, Electrochemistry, Materials for devices, Nanoscale materials

## Abstract

BiFe_0.9_Co_0.1_O_3_ is a promising material for an ultra-low-power-consumption nonvolatile magnetic memory device because local magnetization reversal is possible through application of an electric field. Here, changes in ferroelectric and ferromagnetic domain structures in a multiferroic BiFe_0.9_Co_0.1_O_3_ thin film induced by “water printing”, which is a polarization reversal method involving chemical bonding and charge accumulation at the interface between the liquid and the film, was investigated. Water printing using pure water with pH = 6.2 resulted in an out-of-plane polarization reversal from upward to downward. The in-plane domain structure remained unchanged after the water printing process, indicating that 71° switching was achieved in 88.4% of the observation area. However, magnetization reversal was observed in only 50.1% of the area, indicating a loss of correlation between the ferroelectric and magnetic domains because of the slow polarization reversal due to nucleation growth.

## Introduction

Multiferroic materials have multiple ferroic orders such as ferroelectricity, ferromagnetism and ferroelasticity. A combination of ferroelectricity and ferromagnetism has attracted great attention because it would offer a means to control the magnetization through application of an electric field and possibly make an electric-field-driven nonvolatile magnetic memory^1,2^. So far, electric-field control of magnetism has been achieved at low temperature in Dy_0.75_Gd_0.25_FeO_3_^3^ and (LuFeO_3_)_*m*_/(LuFe_2_O_4_)_1_^4^, but no direct observation of magnetization reversal by applying an electric field at room temperature was reported before our study^5^. One of the most studied multiferroic materials to date is BiFeO_3_ (BFO), which has robust ferroelectricity with a ferroelectric Curie temperature of 1123 K and antiferromagnetism with a Néel temperature of 643 K^6–10^. BFO has an electric polarization in the [001] direction of a hexagonal *R*3*c* unit cell, which is in the [111] direction in the pseudocubic notation. The spin-5/2 state of the Fe^3+^ ion has a cycloidal spin modulation with a period of 620 Å in the [110] direction in the hexagonal notation superimposed on the G-type antiferromagnetic ordering, resulting in zero spontaneous magnetization^7^. Note that despite the different origins of the ferroelectricity and magnetism in BFO, they are coupled through the Dzyalosihnskii-Moriya interaction^11,12^ induced by the rotation of the FeO_6_ octahedron, and control of magnetism by using an electric field has been achieved in both bulk^13^ and thin films^14^. It has also been reported that the magnetization of a ferromagnetic material bonded to a BFO thin film can be controlled by applying an electric field to the BFO^14,15^.

We reported that partial substitution of Co with Fe removed the spin cycloidal modulation and stabilized a canted collinear spin structure with a spontaneous magnetization perpendicular to the electric polarization^16^. Piezoresponse force microscopy (PFM) and magnetic force microscopy (MFM) observations in BiFe_0.9_Co_0.1_O_3_ thin films grown in the (001)_pc_ orientation on a (110)_o_-oriented GdScO_3_ (GSO) substrate (“pc” and “o” denote pseudocubic and orthorhombic indices, respectively) showed similar striped ferroelectric and ferromagnetic domain structures, indicating a strong correlation between them. Moreover, local magnetization reversal accompanying a 71° out-of-plane (OOP) polarization reversal was observed^5^. It was also demonstrated that both the ferroelectric and magnetic domain structures can be controlled by the trailing field, which is an electric field induced by moving the cantilever with biased voltage on the surface of the film^17,18^. Thus, BFCO is a promising candidate for electric-field-write and magnetic-read-out nonvolatile memory devices with very low power consumption.

The changes in the ferroelectric and magnetic domains accompanying polarization reversal must be repeatable and deterministic in order to apply this phenomenon to a memory device. However, application of an electric field by moving a biased cantilever on the BFCO film surface (electric-field polarization reversal) leads to surface damage and inhomogeneous polarization reversal because of the high concentration of the electric field, which causes secondary effects such as Joule heating and redox reactions^17,19^. Therefore, perfect OOP polarization reversal can be performed only three times. Applying voltage through metal electrodes deposited on the film surface does not cause such a problem, but the electrode will interfere with the direct domain observations made by PFM and MFM. As a way to avoid these problems, we focused on water printing^20–22^, which is an OOP polarization reversal method involving chemical bonding and charge accumulation at the interface between a liquid and film. This method should be able to apply a uniform electric field and not damage the film surface. By blowing off the liquid solution after water printing, we can directly observe the ferroelectric and magnetic domains by PFM and MFM. Polarization reversal by water printing from upward to downward and vice versa has been already achieved in a BFO/(La,Sr)MnO_3_ (LSMO) system^22^. In this study, we used this method to reverse the polarization in a BFCO thin film and observed the changes in the domain structures accompanying OOP polarization reversal by using PFM and MFM.

## Methods

### Thin film fabrication

BFCO (40 nm) thin films were grown on (110)_o_-oriented GSO single crystal substrates by pulsed laser deposition (PLD) with a KrF excimer laser (*λ* = 248 nm). SrRuO_3_ (SRO) (20 nm) was fabricated on the GSO as a bottom electrode prior to deposition of the BFCO film. GSO substrates were cleaned by ultrasonification with ethanol and acetone and were annealed at 1000 °C for 4 h in the air before film deposition. The substrates had atomically smooth surfaces after these processes. The substrate temperatures during growth of the BFCO and SRO were set to 604–608 °C and 667 °C, and the oxygen pressures were 13 Pa and 15 Pa, respectively. The PLD targets were of stoichiometric ratio. After the deposition process, the films were cooled to room temperature over a period of about 40 min at an oxygen partial pressure of 5000 Pa to improve the insulating properties^17^.

### Characterizations

The crystal structure and crystallinity of the BFCO thin films were investigated using X-ray diffraction (XRD) (Rigaku SmartLab). Surface topography, ferroelectric and magnetic domains were observed using contact-mode atomic force microscopy (AFM), PFM and MFM (Asylum Research Cypher S). The AFM and PFM cantilevers were ASYLEC.01-R2 (OXFORD INSTRUMENTS) and the MFM cantilever was MFMR (NANOWORLD). Angle-resolved X-ray photoelectron spectroscopy (ARXPS) (ULVAC-PHI, INC. PHI 5000 VersaProbe III) with an Al Kα (*hν* = 1486.6 eV) photon source was used to determine the surface termination of the film. Eight polarization directions were identified using one OOP and two in-plane (IP) PFM phase images, and 3D-PFM images^5^ were constructed with eight colors so that all polarization directions could be distinguished. To confirm the magnetic origin of the MFM contrast, we reversed the magnetization of the cantilever and confirmed the reversal of the contrast.

## Results and discussion

**Figure 1:**
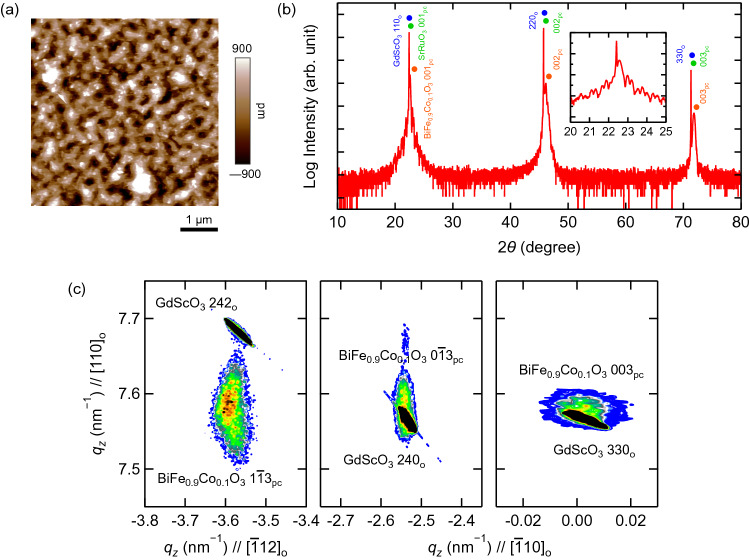
(**a**) BFCO surface topography investigated by contact-mode AFM. (**b**) XRD *ω*–2*θ* pattern of BFCO/SRO/GSO thin film. The inset shows the Laue fringes of BFCO and SRO around the GSO 110_o_ peak. (**c**) RSMs of BFCO/SRO/GSO taken around 330_o_, 240_o_ and 242_o_ reflections of GSO.

Figure 1a shows the BFCO surface topography investigated by contact-mode AFM. The root mean square of the surface roughness was less than 0.5 nm; thus, the prepared BFCO thin film was very flat. The XRD *ω*–2*θ* pattern of the BFCO/SRO/GSO thin film is shown in Fig. 1b. The XRD pattern exhibits clear 00*h*_pc_ diffraction peaks of BFCO and SRO without peaks from impurities. Laue fringes originating from 40 nm-thick BFCO are superimposed on those from 20 nm-thick SRO with twice the period of BFCO around the GSO 110_o_ peak. Therefore, these two films were epitaxially grown and had very smooth surfaces^23^. Reciprocal space maps (RSMs) of BFCO/SRO/GSO taken around 330_o_, 240_o_ and 242_o_ reflections of GSO are shown in Fig. 1c. These maps indicate that the BFCO films were coherently grown on the GSO due to the small mismatch. The $0\overline{1}3_{{{\text{pc}}}}$ peak split into two and the $1\overline{1}3_{{{\text{pc}}}}$ peak split into three, indicating that the crystal structure of BFCO was a nearly rhombohedral-like monoclinic one with polarization in the [111]_pc_ direction, the so-called *M*_*A*_ phase in previous studies^24,25^. The SRO peak is not visible in the RSMs because of the overlapping GSO peak. The pseudocubic lattice parameters of BFCO are *a*_pc_ = 3.94 Å, *b*_pc_ = 3.96 Å, *c*_pc_ = 3.96 Å and *β*_pc_ = 89.6°.

**Figure 2:**
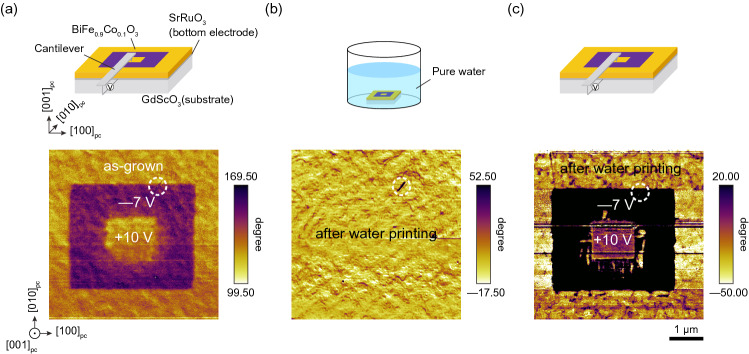
(**a**) OOP PFM phase image of BFCO/SRO/GSO (5 × 5 µm^2^) after polings at − 7 V (3 × 3 µm^2^) and at + 10 V (1 × 1 µm^2^). (**b**) OOP PFM phase image of the same area after water printing in pure water (pH = 6.2) for about 5 h. (**c**) OOP PFM phase image in the same area after poling with the voltage applied in the same way as shown in (**a**).

Figure 2a shows an OOP PFM phase image of BFCO/SRO/GSO (5 × 5 µm^2^) obtained after poling of the 3 × 3 µm^2^ area by scanning with the PFM cantilever with a –7 V bias voltage followed by poling of the central 1 × 1 µm^2^ area with a + 10 V bias voltage. The contrasts of the as-grown state in the outermost area and the reversed state revealed by the − 7 V poling in the 3 × 3 µm^2^ area are opposite, indicating that all polarizations in the as-grown state point downward. This is due to the SrO-terminated surface of the bottom electrode SRO^26,27^. The clear contrasts indicate that the BFCO thin film was sufficiently insulating to enable at least two polarization reversals.

Next, the BFCO thin film was immersed in pure water (pH = 6.2 at room temperature) for about 5–10 h. The slightly acidic pH of pure water is due to the absorption of CO_2_ from the air. Figure 2b shows an OOP PFM phase image obtained after blowing off the pure water with an N_2_ gun. The contrast after water printing was uniform, indicating successful poling in the entire area, either upward or downward. To determine the polarization direction after water printing, we performed electric-field poling in the same way as in Fig. 2a. The result shown in Fig. 2c has the same contrast as in Fig. 2a, indicating that the polarization direction after water printing was the same as in the as-grown state, downward. A scratch on the thin film surface circled by the dashed line indicates that all the PFM images were taken in the same area. The corresponding contact-mode AFM images of Fig. 2b, c are shown in Fig. S1; there is no change in surface morphology before and after water printing, indicating that the BFCO surface was not affected by this process.

**Figure 3:**
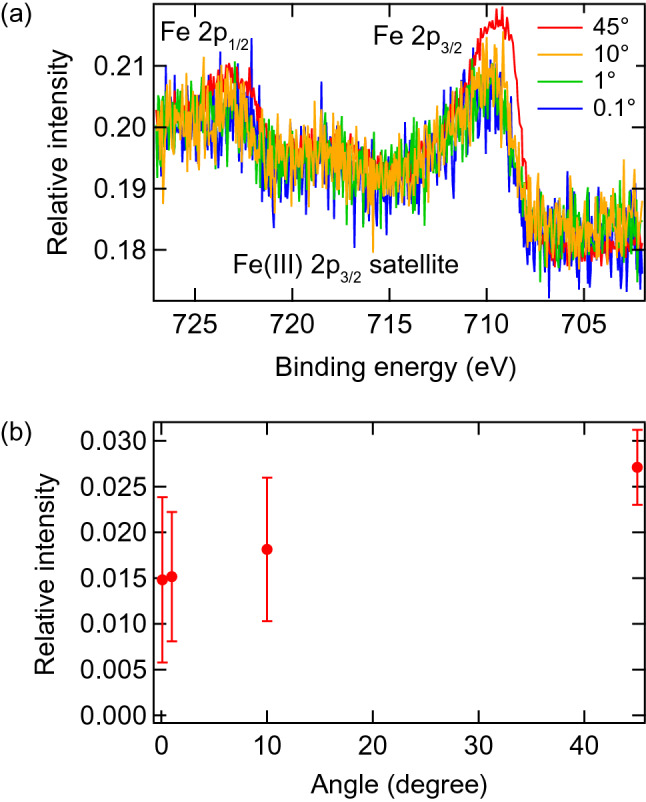
(**a**) Fe 2*p*_1/2_ and Fe 2*p*_3/2_ spectra obtained at various detection angles normalized to the Bi 4*f*_5/2_ peak height. (**b**) Detection angle dependence of the Fe 2*p*_3/2_ peak intensity.

In previous studies on BFO^22^, water printing in acidic solutions with the pH lowered to 3 was necessary to reverse the polarization from upwards to downwards. In BFCO/SRO/GSO, however, the out-of-plane polarization reversal from upwards to downwards was achieved even in acidic solutions with a pH close to 7. Because the surface termination of the thin film determines the chemical bonding and the sign of the charge accumulated in water printing, we investigated the composition of the termination surface by ARXPS and clarified the mechanism of water printing in the BFCO/SRO/GSO system. Figure 3a shows the Fe 2*p*_1/2_ and Fe 2*p*_3/2_ spectra obtained at various detection angles between the sample surface and the detector. The plots are normalized to the height of the Bi 4*f*_5/2_ peak. The raw data of the Fe 2*p*_1/2_, Fe 2*p*_3/2_, Bi 4*f*_5/2_ and Bi 4*f*_7/2_ spectra are shown in Fig. S2. Since the smaller detection angle provides information from the shallower part reflecting the surface structure and the detection depth is approximately proportional to the sine of the detection angle, the detection depths compared with that at a 45-degree detection angle (estimated to be 3 − 4 nm for the photon energy of Al Kα) are about 1/4 at 10 degrees, 1/40 at 1 degree and 1/400 at 0.1 degrees. No shift was found in the Fe 2*p*_3/2_ satellites, indicating the valence state of Fe was homogeneously 3 + between the surface and the interior of the film. It is clear that the normalized Fe 2*p*_3/2_ intensities at smaller angles are smaller than that at 45 degrees. We determined the intensity of the Fe 2*p*_3/2_ peak by making a Gaussian fit and the result is plotted as a function of the detection angle after subtracting the background in Fig. 3b. The intensity of the Fe 2*p*_3/2_ peak becomes smaller as the detection angle decreases below the error, including both the measurement and fitting analysis errors, which indicates that the amount of Fe was smaller than that of Bi at the BFCO surface and the surface termination was a BiO plane. It is known that BFO grown on the SRO bottom electrode has an FeO_2_ layer above the SrO-terminated surface of SRO^26,27^. It is also reported that when growth of BFO in an O-rich environment starts from an FeO_2_ plane, the termination is a BiO plane^28^. Accordingly, our finding of BiO surface termination in BFCO/SRO/GSO is reasonable. The presumed mechanism of water printing in the present BFCO/SRO/GSO system is as follows. The binding between the H^+^ in the slightly acidic pure water and the O in the BiO plane causes positive charge to accumulate on the surface of the BFCO film and an OOP polarization reversal occurs to shield the accumulated charge. The water printing in an alkali solution that was observed for BFO/LSMO did not occur in the present BFCO/SRO/GSO system because OH^–^ ions could not bond to the BiO layer. It has also been reported that the BiO-terminated surface is reconstructed so that more oxygen atoms are present on the topmost surface when fabrication is carried out under oxygen-rich conditions^29^. Since our BFCO films were cooled down to room temperature at an oxygen partial pressure of 5000 Pa, it is reasonable to suppose that O at the BiO-terminated surface prohibited the bonding of OH^−^ and polarization reversal in an alkaline solution. These results indicate that polarization reversal by water printing is sensitive to the surface termination of the film.

**Figure 4:**
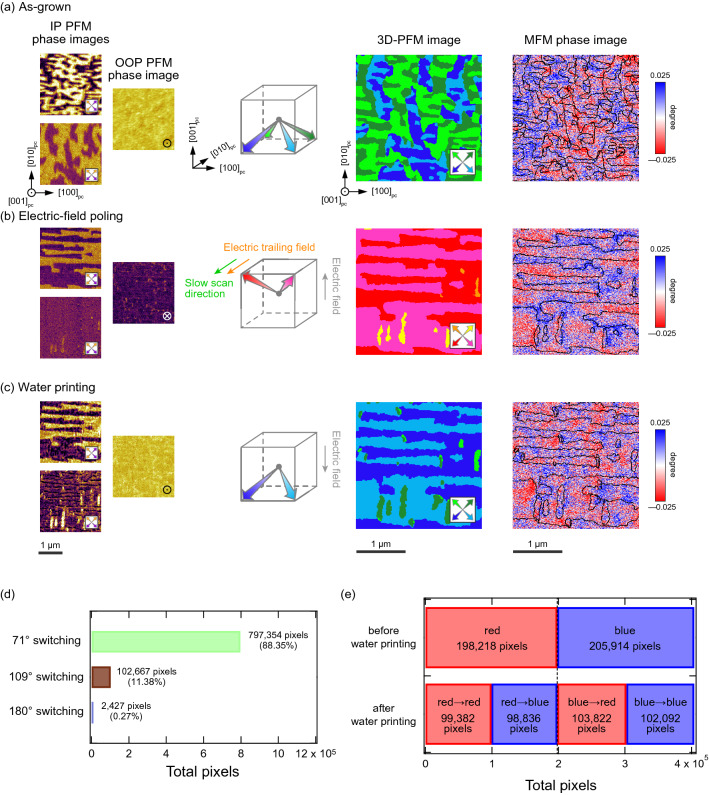
Changes in ferroelectric and magnetic domain structures of BFCO film due to electric-field poling followed by water printing: (**a**) as-grown state, (**b**) after electric-field poling with a − 7 V bias voltage and (**c**) after subsequent water printing for 10 h. The leftmost figures are PFM phase images identifying the polarization component in the [100]_pc_ (IP), [010]_pc_ (IP) and [001]_pc_ (OOP) directions. The IP PFM image on the upper (lower) side identifies the horizontal (vertical) polarization direction. The arrows in the lower right of the PFM images indicate the directions of the polarizations corresponding to the contrast color. The schematic diagrams second from the left show the polarization directions in a BFCO pseudocubic unit cell, which correspond to the colors of the ferroelectric domains in the 3D-PFM images. In these diagrams, “slow scan direction,” “electric trailing field” and “electric field” mean the slow scan direction of the cantilever, the direction of the electric trailing field and the direction of electric field between the bottom electrode and the cantilever. The 3D-PFM images third from the left were constructed by superimposing the two IP and OOP PFM phase images. The rightmost images are MFM phase images with the ferroelectric domain boundary (black line) of the same observation area as the 3D-PFM images. (**d**) Numbers of pixels in 71°, 109° and 180° switching areas. (**e**) Numbers of pixels that changed to their opposite color in MFM images before and after water printing.

As OOP polarization of a BFCO film by water printing was demonstrated, we investigated how the IP ferroelectric domain structure was affected. First, we reversed the downward polarization of the as-grown film by electric-field poling; then, we reversed the polarization from upwards to downwards by water printing. Figure 4a–c respectively show the ferroelectric and magnetic domains in the as-grown state, after poling by scanning with the cantilever with a –7 V bias voltage and after water printing using pure water (pH = 6.2). The leftmost panels are PFM phase images of the BFCO film identifying the polarization component in the [100]_pc_ (IP), [010]_pc_ (IP) and [001]_pc_ (OOP) directions. The IP PFM image on the upper (lower) side identifies the horizontal (vertical) polarization direction. The arrows in the lower right of the PFM images indicate the direction of polarization corresponding to the contrast color.

Comparing the OOP PFM phase images before and after the electric-field poling in Fig. 4a, b, it can be seen that the contrasts are uniformly reversed, indicating that OOP polarization reversal was achieved in the entire observation area. This is also evident from the OOP PFM images including the unwritten surrounding area shown in Fig. S3. On the other hand, the IP ferroelectric domain structure changed into a horizontal stripe one in most of the area because of the trailing field. It should be noted that the electrostatic and elastic energies are minimized in a striped domain structure^30–32^. 3D-PFM images constructed by combining the two IP and one OOP PFM phase images are shown in the third column from the left in Fig. 4a–c. All eight polarization directions can be distinguished from these images. The schematic illustrations to the left of the 3D-PFM images exhibit the polarization directions in the BFCO pseudocubic unit cell corresponding to the colors of the ferroelectric domains in the 3D-PFM images. In these illustrations, “slow scan direction,” “electric trailing field” and “electric field” indicate the slow scan direction of the cantilever, the direction of the electric trailing field^18,33–36^ and the direction of the electric field between the bottom electrode and the cantilever. The four IP polarization directions in the 3D-PFM image in Fig. 4a are reduced to two (indicated in red and pink in Fig. 4b) by the trailing field, although there is a small area (indicated in yellow and orange) where the IP polarization is antiparallel to the trailing field. To the right of the 3D-PFM image is the MFM phase image with the ferroelectric domain boundary (black line). The magnetic origin of the MFM contrast was confirmed by reversing the magnetization of the cantilever, as shown in Fig. S4. A clear correlation between the ferroelectric and magnetic domains is evident in Fig. 4b, but the domains are slightly shifted relative to each other. This shift is because of the difference in surface sensitivity between the MFM and PFM methods and the inclination of the 71° ferroelectric domain wall relative to the film normal, as previously reported^5^.

The contrast of the OOP PFM image in Fig. 4c is reversed from that in Fig. 4b; this indicates that the OOP component of the polarization changed from upward to downward by water printing, as observed in Fig. 2. The ferroelectric domain structure in the IP PFM images showed almost no change after water printing, indicating that a 71° polarization reversal where magnetization reversal was expected was achieved. However, 109° switching was also observed, as evidenced by the red to yellowish green and pink to green color changes in the 3D-PFM images in Fig. 4b, c. The MFM phase image in Fig. 4c also changed from that in Fig. 4b, but the correlation to the 3D-PFM image was less clear after water printing. We quantitatively evaluated the ratio of the areas where 71°, 109° and 180° switching occurred and the OOP component of magnetization was reversed after water printing by counting the pixels with the corresponding color changes in the PFM and MFM images (Fig. S5). The results are plotted in Fig. 4d, e. We found that a 71° polarization reversal was achieved in 88.4% of the observed area by water printing, but magnetization was reversed in only 50.1% of these regions. In other words, the correlation between the ferroelectric and magnetic domains was lost. This result is in contrast to the 71° polarization switching caused by electric-field poling where the magnetization reversal preserving the striped domain structure was observed^5^.

Neutron diffraction and Mössbauer spectroscopy studies have found that the antiferromagnetic spin directions are perpendicular to the polarization in BFCO and the spontaneous magnetization owing to the Dzyalosihnskii-Moriya interaction generated by the octahedral tilting is perpendicular to both the polarization and spin directions^5,19,37,38^. Mössbauer spectroscopy on the thin film also revealed that the spin direction was limited to four out of six <$\overline{1}21$>_pc_ directions, as shown in Fig. S6. A change in the spin direction to other possible directions is necessary for the reversal of the OOP component of the magnetization accompanying the 71° polarization reversal. In the present water-printing experiment, the correlation between the ferroelectric and magnetic domains was lost because there were regions where the OOP component of the magnetization was reversed and not reversed even in one ferroelectric domain. This should be attributed to the slow polarization reversal caused by water printing with inhomogeneous nucleation of small ferroelectric domains. Even though 71° polarization switching was eventually achieved in the entire region, the magnetic domain was divided into smaller regions and the magnetization reversal did not occur in half of them.

## Conclusion

In conclusion, we investigated the changes in the ferroelectric and magnetic domain structures of BFCO/SRO/GSO after water printing. Water printing with pure water (pH = 6.2) led to 71° polarization switching over the entire film where OOP polarization was reversed from upward to downward while the IP polarization direction was preserved. This happened because of an accumulation of positive charge of H^+^ binding to O in the BiO plane termination surface of BFCO/SRO/GSO, as determined by our ARXPS measurement. Quantitative analysis of 3D-PFM and MFM images revealed that the 71° polarization reversal was achieved in 88.4% of the observed regions while reversal of the OOP component of magnetization occurred in only 50.1% of the corresponding regions, resulting in a loss of correlation between the ferroelectric and magnetic domains. We believe that the slow polarization reversal in water printing with inhomogeneous nucleation of small ferroelectric domains divided the magnetic domains into smaller ones and the magnetization reversal did not occur in half of the smaller domains.

## Supplementary Information

Supplementary Information.

## Data Availability

The data used and/or analyzed during the current study are available from the corresponding authors upon reasonable request.
