# Supplementary material for: Out-of-plane polarization reversal and changes in in-plane ferroelectric and ferromagnetic domains of multiferroic BiFe0.9Co0.1O3 thin films by water printing
Source: Sci Rep. 2023 May 4;13:7236. doi: 10.1038/s41598-023-34386-3 (PMC10160096; doi:10.1038/s41598-023-34386-3)
Supplement: Supplementary file 1 — Supplementary Information. [file 41598_2023_34386_MOESM1_ESM.pdf]

Supplementary Information

Out-of-plane polarization reversal and changes in in-plane ferroelectric and ferromagnetic domains of multiferroic $BiFe_{0.9}Co_{0.1}O_3$ thin films by water printing

Takuma Itoh[1*], Kei Shigematsu[1,2*], Takumi Nishikubo[1,2], Masaki Azuma[1,2,3]

[1]Laboratory for Materials and Structures, Institute of Innovative Research, Tokyo Institute of Technology, Yokohama, Kanagawa 226-8503, Japan

[2]Kanagawa Institute of Industrial Science and Technology, Ebina, Kanagawa 243-0435, Japan

[3]Living Systems Materialogy Research Group, International Research Frontiers Initiative, Tokyo Institute of Technology, Yokohama 226-8501, Japan

Surface morphology before and after water printing

Figures S1 (a) and (b) show the contact-mode AFM images of the BFCO film surface obtained after poling by scanning with the biased cantilever and after water printing, respectively. There is no change in surface morphology in the two AFM images, indicating that the BFCO surface is not affected by the poling processes.

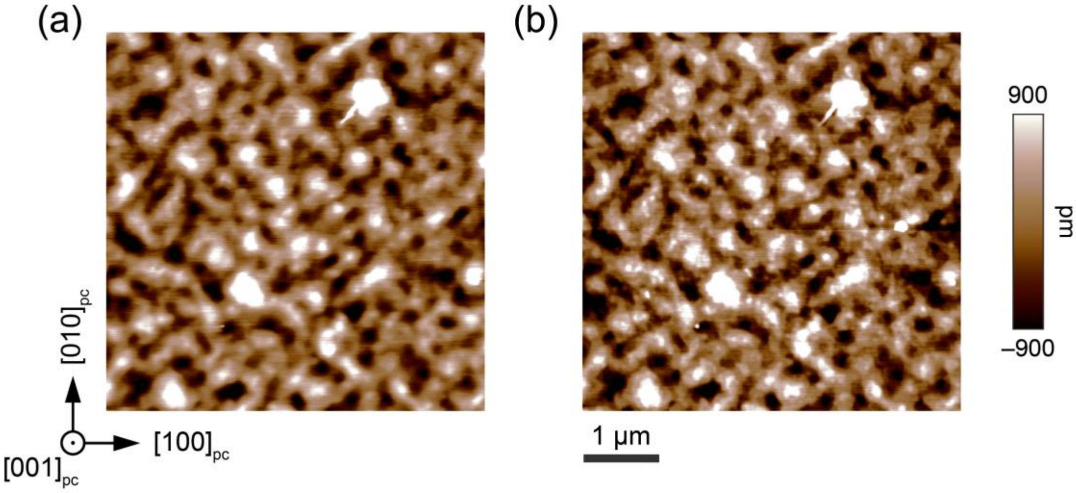

Figure S1. (a) AFM image of BFCO/SRO/GSO (5 × 5 μm^2) after polings at –7 V (3 × 3 μm^2) and at +10 V (1 × 1 μm^2). (b) AFM image of the same area after water printing in pure water (pH = 6.2) for about 5 hours.

Raw ARXPS spectra of BFCO/SRO/GSO thin film

The Bi 4f and Fe 2p ARXPS spectra (counts per second, cps vs. binding energy) of BFCO/SRO/GSO obtained at various detection angles (0.1°, 1°, 10°, 45°) between the sample surface and the detector are shown in Figs S2 (a) and (b). In both measurements, the intensity decreases as the detection angle decreases, but the decrease rate of the intensity of the Fe $2p_{3/2}$ peak determined by a Gaussian fit after subtracting the background is larger than that of Bi 4f, indicating that the surface termination is BiO.

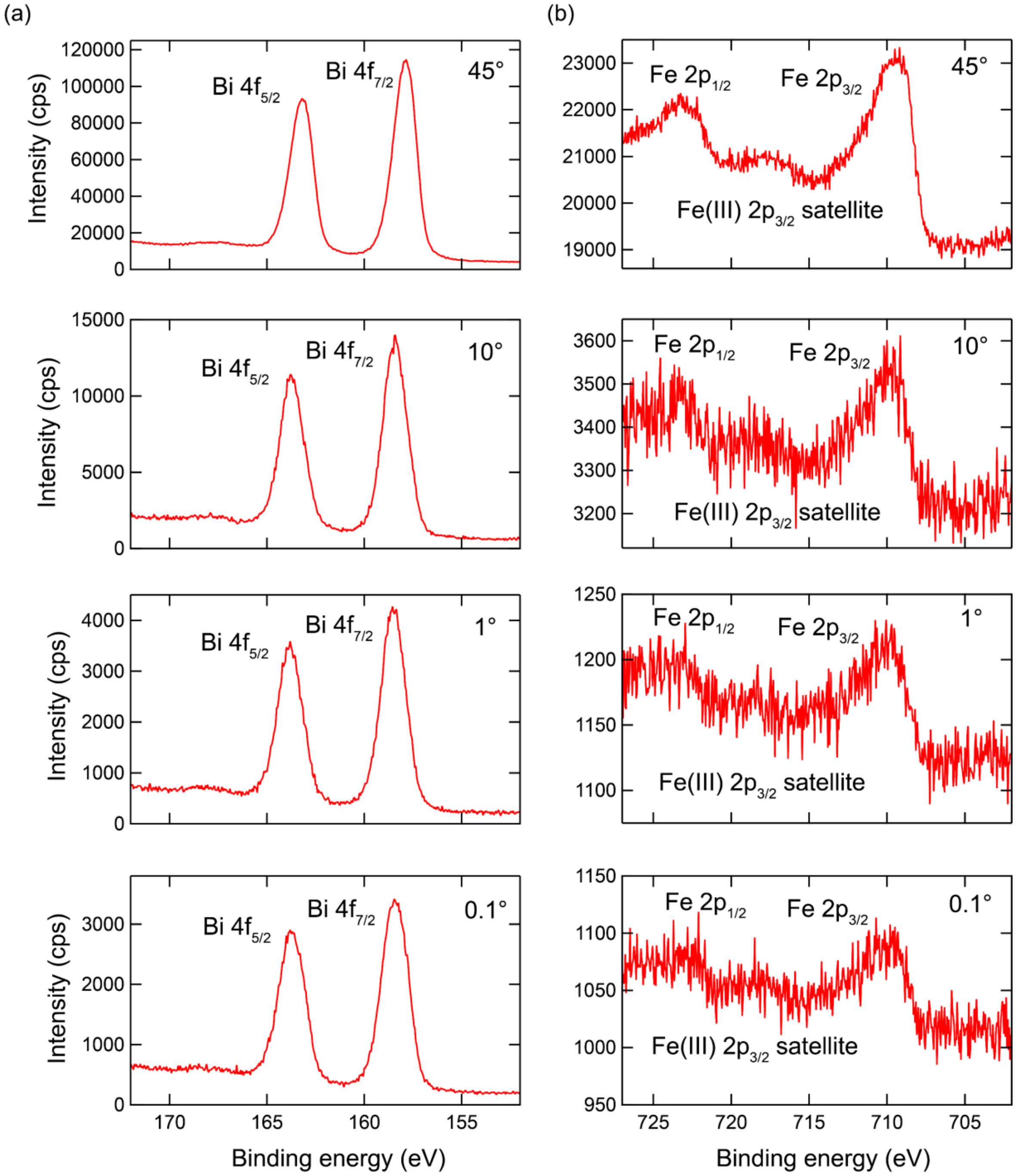

Figure S2. (a) Bi 4f and (b) Fe 2p in BFCO spectra obtained at various detection angles.

OOP polarization reversal of BFCO thin film using electric-field poling and water printing

Figure S3 shows the OOP PFM phase images including the unwritten surrounding area of Fig. 4. Figures S3 (a), (b) and (c) are in the as-grown state, after poling by scanning the cantilever with a −7 V bias voltage and after water printing using pure water (pH = 6.2), respectively. The contrast became uniform again after water printing, indicating that OOP polarization was successfully reversed.

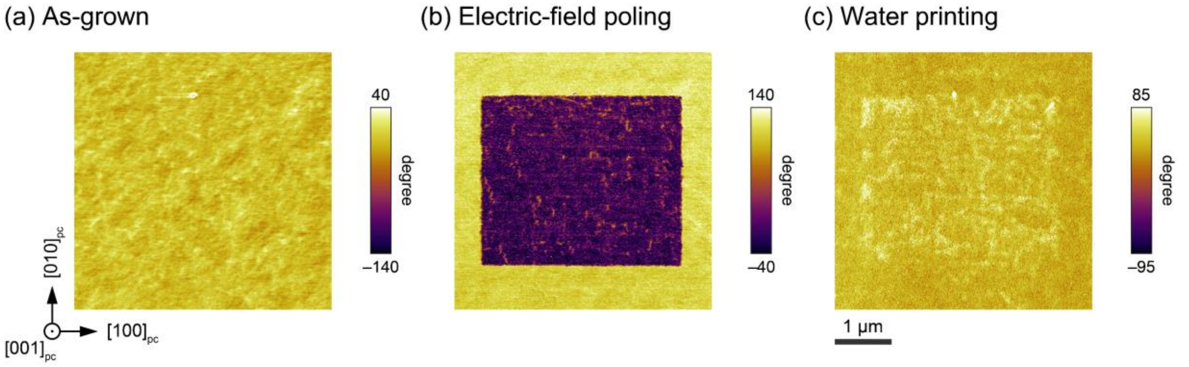

Figure S3. PFM phase images identifying the polarization component in the $[001]_{pc}$ direction (OOP): (a) as-grown state, (b) after electric-field poling with a −7 V bias voltage and (c) after water printing.

Reversal of MFM contrast by reversal of MFM tip magnetization

Figure S4 (a) is the same MFM phase image as Fig. 4 (b). The image obtained after the reversal of the cantilever tip magnetization is shown in Fig. S4 (b). The areas with blue color and with red color are reversed in the entire area, confirming the magnetic origin of the MFM signal.

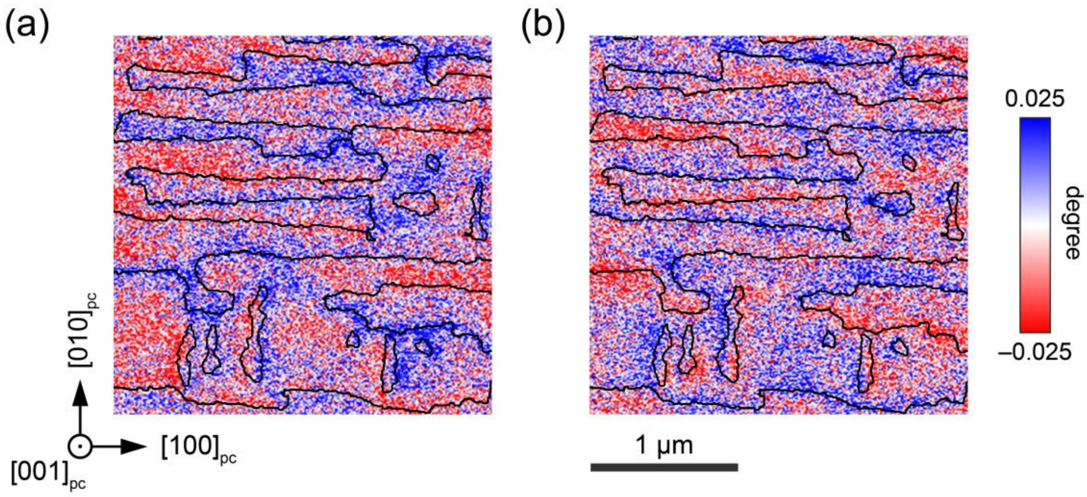

Figure S4. (a) MFM phase image after electric-field poling (same as Fig. 4 (b)). (b) MFM phase image after reversal of MFM cantilever tip magnetization. Black lines show the ferroelectric domain boundary.

Details of quantitative PFM and MFM images analysis

Figure S5 (a) shows the locations of the 71° switching (red to blue, pink to light blue, yellow to green and orange to yellowish green color changes in the 3D-PFM images in Figs. 4 (b) and (c)), 109° switching (red to yellowish green, red to light blue, pink to green, pink to blue and yellow to light blue) and 180° switching (red to green and pink to yellowish green) after water printing. The fraction of each area determined by counting the pixels of the corresponding color change is plotted in Fig. 4 (d). We also quantitatively evaluated the fraction of the area where the 71° polarization switching accompanied the OOP magnetization reversal. First, the MFM image before water printing shown in Fig. S5 (b) was shifted upward by 20 pixels so that the ferroelectric domain boundary determined from the PFM image in Fig. 4 (b), indicated with solid line, matched the magnetic domain boundary because these two boundaries are shifted with each other owing to the inclined ferroelectric domain wall and difference in the depth sensitivity of PFM and MFM, as shown in Ref. 3. The MFM image after water printing was also shifted by the same amount as shown in Fig. S5 (c). Note that the positions of the vertical striped domains cannot be matched. We believe this is because of the different inclining directions. The area surrounded by the dashed line in Fig. S5 (b) was therefore removed from further analysis. The 109° and 180° switching areas were also removed and the resultant MFM images are presented in Fig. S5 (d).

Finally, the pixels of each color change (red to red, red to blue and vice versa) were counted and are plotted in Fig. 4 (e). 50.1% of the area among the 71° polarization switching area (88.4% of the entire observation area) exhibited magnetization reversal, indicating a loss of correlation between the ferroelectric and magnetic domains.

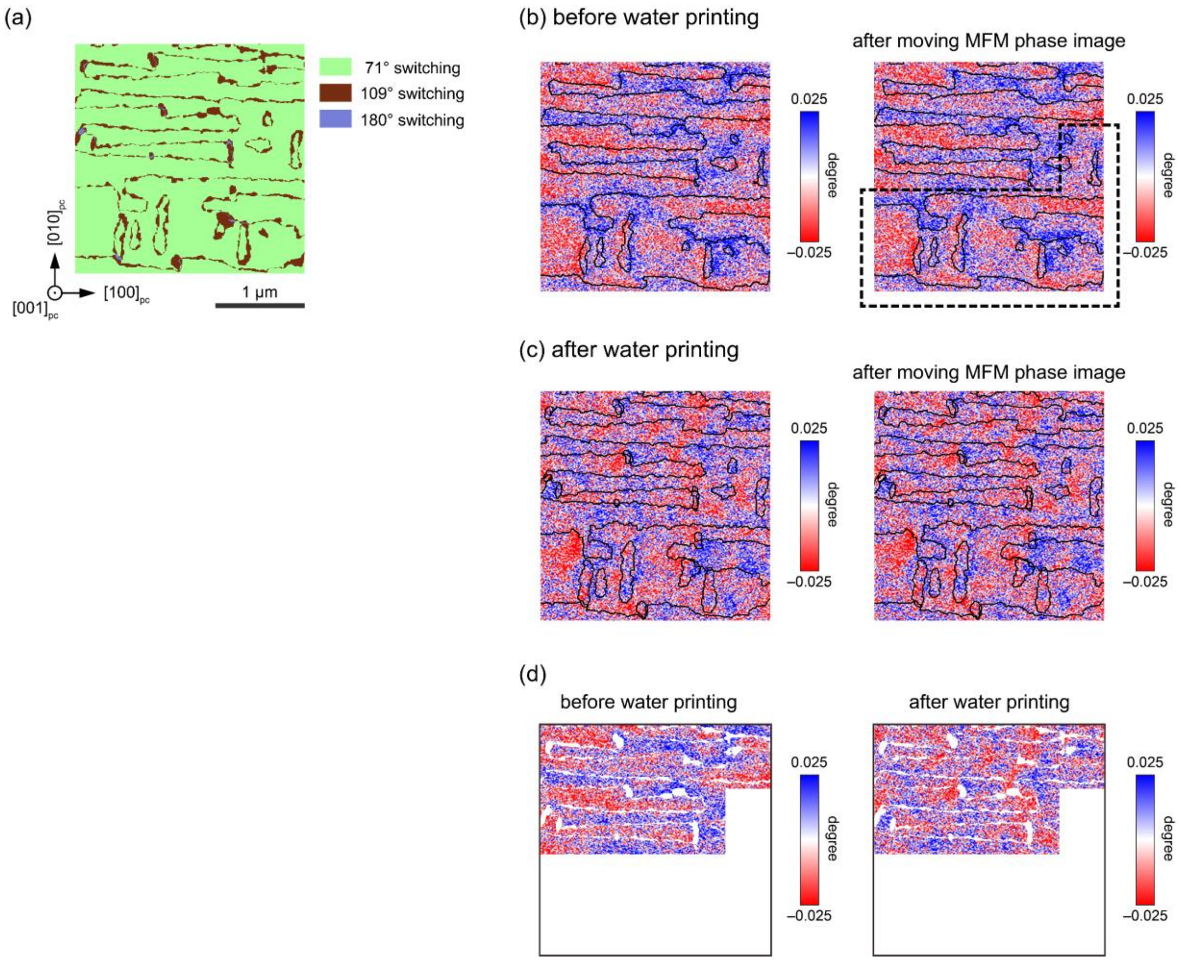

Figure S5. Determination of the area fraction where the OOP magnetization reversal accompanying 71° polarization switching was achieved by water printing. (a) Areas of the 71°, 109° and 180° polarization reversals by water printing determined by comparing Figs. 4 (b) and (c). (b) MFM phase images after poling with the cantilever with –7 V, before (left) and after (right) the ferroelectric domain wall and magnetic domain were matched. (c) MFM phase images after water printing, before (left) and after (right) the ferroelectric domain wall and magnetic domain were matched. (d) MFM images after poling and after water printing, which was used for quantitative analysis of the magnetization reversal region.

Possible magnetization switching processes accompanying OOP 71° polarization switching

Figure S6 (a) shows the possible four spin vectors (***L***) directions in the easy plane perpendicular to the $<11\bar{1}>_{pc}$ ***P*** direction determined by the detailed analysis of Mössbauer spectroscopy, as described in Ref. 3. These correspond to four out of six $<\bar{1}21>_{pc}$ directions. No magnetization reversal takes place when the spin vector changes from $\boldsymbol{L_1'}$ to $\boldsymbol{L_1''}$ or $\boldsymbol{L_2''}$ in Fig. S6 (b). Reversal of OOP magnetization takes place when the spin vector changes from $\boldsymbol{L_1'}$ to $\boldsymbol{L_3''}$ or $\boldsymbol{L_4''}$.

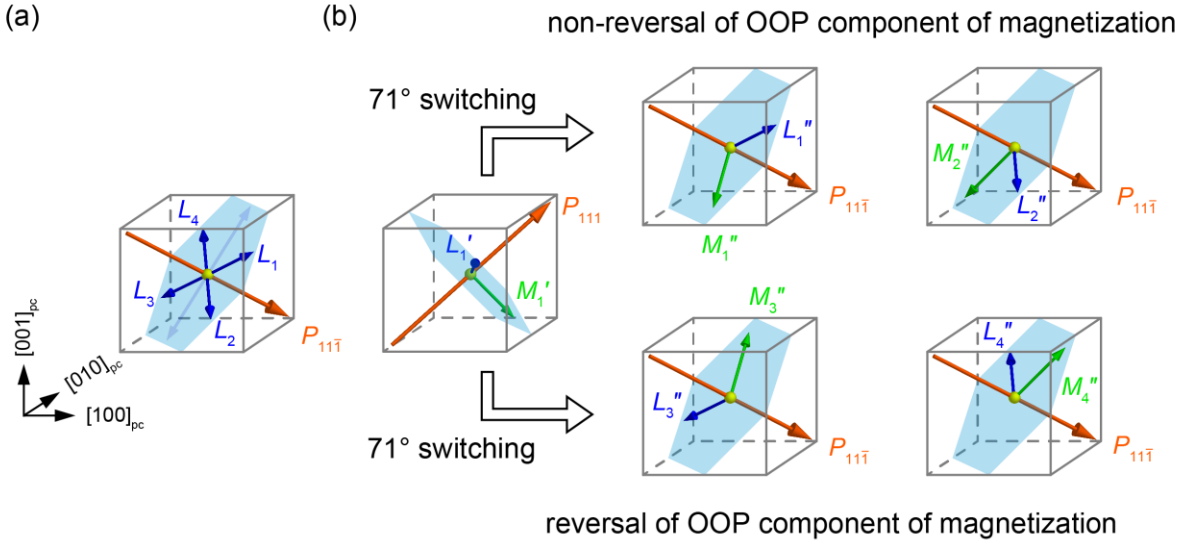

Figure S6. (a) Possible spin direction $\boldsymbol{L}_i$ *(i* =1–4) in BFCO/SRO/$GdScO_3$ $(110)_o$ thin film. (b) Changes in spin direction ***L*** and magnetization ***M*** after polarization reversal induced by an electric field.
